# The Construct of the Schizophrenia Quality of Life Scale Revision 4 for the Population of Taiwan

**DOI:** 10.1155/2017/5328101

**Published:** 2017-01-31

**Authors:** Chia-Ting Su, Ai-Lun Yang, Chung-Ying Lin

**Affiliations:** ^1^Department of Occupational Therapy, College of Medicine, Fu Jen Catholic University, New Taipei City, Taiwan; ^2^Department of Sports Sciences, University of Taipei, Taipei, Taiwan; ^3^Department of Rehabilitation Sciences, Faculty of Health and Social Sciences, The Hong Kong Polytechnic University, 11 Yuk Choi Road, Hung Hom, Hong Kong

## Abstract

This study examines the factor structure of the Schizophrenia Quality of Life Scale Revision 4 (SQLS-R4) for inpatients with schizophrenia in a psychiatric hospital in southern Taiwan. All the participants (*n* = 100) filled out the SQLS-R4, Mini Mental Status Examination (MMSE), and Brief Psychiatric Rating Scale (BPRS) under the supervision of one experienced occupational therapist. Using confirmatory factor analysis, we first determined that a 29-item model was more satisfactory than the original 33-item model based on the findings of better fit indices for the 29-item model. We then found that a three-correlated-factor structure was best for the SQLS-R4 after four models (namely, two-correlated-factor, three-correlated-factor, seven-correlated-factor, and second-order models) had been compared. In addition, the three constructs (psychosocial, physical, and vitality) were moderately to highly correlated with the constructs of the World Health Organization Quality of Life- (WHOQOL-) BREF (*r* = −0.38 to −0.69), except for one low correlation between the vitality construct of the SQLS-R4 and the psychological construct of the WHOQOL-BREF (*r* = −0.26). We tentatively conclude that the SQLS-R4 with a three-correlated-factor structure is a valid and reliable instrument for examining the quality of life of people with schizophrenia.

## 1. Introduction

Quality of life (QoL) measures have become more important for therapeutic interventions and clinical decisions [1, 2], especially for patients with chronic illness [3]. When people with schizophrenia confront their chronic disabling illness, it is important for healthcare professionals to understand their QoL. QoL, a subjective perception of an individual's health position within their cultural context and value system [4], can be used as one of the long-term goals of medical interventions [3].

Two kinds of QoL measures can be applied to people with schizophrenia: generic QoL measures and schizophrenia-specific QoL measures [5]. Although the schizophrenia-specific QoL measures cannot compare QoLs between different populations with disabilities (e.g., people with stroke and people with spinal cord injury), they focus on the core symptoms that people with schizophrenia encounter [5]. Therefore, the schizophrenia-specific QoL measures are particularly useful for understanding how the symptoms affect the QoL of people with schizophrenia.

Several schizophrenia-specific QoL measures have been developed, and the Schizophrenia Quality of Life Scale Revision 4 (SQLS-R4) has been suggested to be one of the most useful ones [5–7]. In addition to its items specifically designed for people with schizophrenia [5, 8], its other strengths include (1) practical and short content to fill out (10–15 minutes to complete); (2) improved psychometric properties after several revisions [6, 9]; (3) solid factor structure and internal reliability [7–12]; and (4) availability of cross-cultural comparisons by providing rigorously translated versions in 52 languages through standardized procedures: forward translation, reconciliation, and back translation [9].

However, as a newly developed QoL measure [8, 11], the construct of SQLS-R4 still needs to be confirmed. Although the SQLS-R4 has good psychometric properties [6, 10, 13], to the best of our knowledge, only one UK study [7] has used confirmatory factor analysis (CFA) to examine the construct of SQLS-R4. The advantage of using CFA is being able to compare several proposed models and to clarify the construct of the tested instruments [14]. Therefore, the CFA is suitable for understanding the constructs of measurements that are under development, such as the SQLS-R4. Although the SQLS-R4 was suggested to be two-dimensional [6, 7, 12, 13], Martin and Allan [7] indicated that some CFA fit indices are not acceptable (root mean square error of approximation [RMSEA] = 0.11 and weighted root mean square residual [WRMR] = 0.94). In addition, one Singaporean study [11] mentions that the cultural difference between Asia and the West may influence the psychometric properties of the SQLS-R4. Thus, the construct of the SQLS-R4 may differ in Asian and Western cultures. Therefore, for better application and interpretation of the SQLS-R4, we suggest that additional studies on the SQLS-R4 construct in Asian populations are needed.

The former version of the SQLS-R4, SQLS, is a 30-item self-reported measure compromising three domains (psychosocial, motivation and energy, and symptoms and side effects) [8]. Several revisions were carried out for the SQLS to improve its psychometric properties, and finally the SQLS-R4 with two factors was developed with different language versions [7, 9], including Taiwanese version [6, 13]. However, when Kuo et al. [6] examined the psychometric properties of the Taiwan version of SQLS-R4, they found that four items had weak corrected item-total correlation with their domains. Therefore, Kuo et al. [13] further removed the four items and used the retained items to do the exploratory factor analysis. They found that the Taiwan version of SQLS-R4 contained seven factors and proposed three dimensions (psychosocial, physical condition, and validity) to include the seven factors. Kuo et al. [13] proposed the physical condition as one of the dimensions because physical domain is a key component for QoL. Hence, current literature suggests different factor structures for the SQLS-R4 according to its item numbers (29 or 33 items) and underlying factors (2, 3, and 7 factors/dimensions).

Five CFA models of the SQLS-R4 construct are proposed in our study. In addition to the two-factor model, we propose three more models based on the findings and suggestions of one Taiwanese study [13]: a three-factor model (psychosocial, physical, and vitality), a seven-factor model (relationships with others, loneliness, depressed thinking, worry, exhaustion status, somatic concern, and vitality), and a second-order model with seven first-order factors (relationships with others, loneliness, depressed thinking, worry, exhaustion status, somatic concern, and vitality) embedded in three second-order factors (psychosocial, physical, and vitality). Moreover, four items have been found to be weak for the SQLS-R4 Taiwan version [13], and, thus, a comparison between a 33-item model and a 29-item model was conducted.

We investigated the SQLS-R4 construct for the population of Taiwan and suggest appropriate items to improve the SQLS-R4's psychometric properties. Specifically, we first compared 33-item, 29-item, three-factor, seven-factor, and second-order models. We then tested the determined underlying constructs for their concurrent validity with one generic QoL measure (World Health Organization Quality of Life [WHOQOL]-BREF).

## 2. Methods

The study was approved by the Institutional Review Board of National Cheng Kung University Hospital, and all the participants filled out and signed informed consents before beginning this study.

### 2.1. Participants and Procedure

Convenience sampling was used to recruit the participants from a psychiatric hospital in southern Taiwan. Like some other mental institutions in Taiwan, this psychiatric hospital was mainly designed to take long-term care of patients with chronic mental illness. Although symptoms remained relatively stable, most of the patients in this hospital were unable to totally live independently in community due to severity of symptoms, insufficient family support, or other issues. All the participants in our study were diagnosed with schizophrenia based on the DSM IV-TR [15] definition, and all were more than 18 years old (age range: 27 to 66). In addition, they all met the inclusion criteria of an illness duration > 2 years, no medication adjustment within the previous two months, a score ≥ 24 on the Mini Mental Status Examination (MMSE), and a score < 24 on the Brief Psychiatric Rating Scale (BPRS). Participants were excluded if they met the following exclusion criteria: (1) impaired cognitive ability to complete the questionnaires as assessed by one occupational therapist with more than 10 years of clinical experience using clinical observation and (2) with comorbid diagnoses of anxiety, depression, bipolar disorder, organic mental disorder, dementia, intellectual disability, and learning disability. All participants were asked to complete several questionnaires mentioned below. Finally, the data of 100 participants, a minimum criterion for performing CFA [16, 17], were used in the study.

### 2.2. Instruments

#### 2.2.1. The Schizophrenia Quality of Life Scale Revision 4 (SQLS-R4)

The SQLS was initially developed to resolve the lack of QoL measures for people with schizophrenia [8]. The original version of the SQLS contains 30 items distributed between three factors: psychosocial (15 items), motivation and vitality (7 items), and symptoms and side effects (8 items). It has a satisfactory internal consistency (*α* = 0.80–0.93) [8]. After several revisions that improved its psychometric properties, the latest version of the SQLS (SQLS-R4) contains 33 items distributed between two factors: psychosocial (20 items) and vitality (13 items) [9]. The SQLS-R4 is self-rated, and all but four items are coded on a 5-point scale in relation to their frequency of occurrence during the previous week: 0 = never and 4 = always. The exceptional 4 items are coded the opposite way: 0 = always and 4 = never. A higher SQLS-R4 score represents a worse QoL.

#### 2.2.2. The WHO Questionnaire on the Quality of Life, Short Form (WHOQOL-BREF)

We used the WHOQOL-BREF Taiwan version, which has 28 items (two items are domestic items, and other items are international items). Four domains (physical health, psychological health, social relations, and environment) are included in the WHOQOL-BREF. The psychometric properties of the WHOQOL-BREF Taiwan version have been tested (e.g., the internal consistency [Cronbach's *α* = 0.70–0.91], the test-retest reliability [*r* = 0.76–0.80], and the construct validity [CFI = 0.89]) and were found to be satisfactory [18]. In addition, a recent study found that the construct of the WHOQOL-BREF Taiwan version fit well with the schizophrenia population and is suitable for using to measure the QoL of people with schizophrenia [19]. A higher WHOQOL-BREF score represents a better QoL.

#### 2.2.3. Mini Mental State Examination (MMSE)

The MMSE contains 11 items and has a total score of 30. It is commonly used to screen the function of cognition; a score ≥ 24 suggests an intact cognition function. The test-retest reliability (*r* = 0.89–0.98) and interrater reliability (*r* = 0.83) have been examined for the Chinese version of the MMSE [20].

#### 2.2.4. Brief Psychiatric Rating Scale (BPRS)

The BPRS contains 16 items; it is designed to measure the severity of psychiatric symptoms. A lower BPRS score indicates a better psychological condition, and the internal consistency of the Chinese version of BPRS is good (Cronbach's *α* = 0.80) [21].

### 2.3. Data Analysis

**Figure 1:**
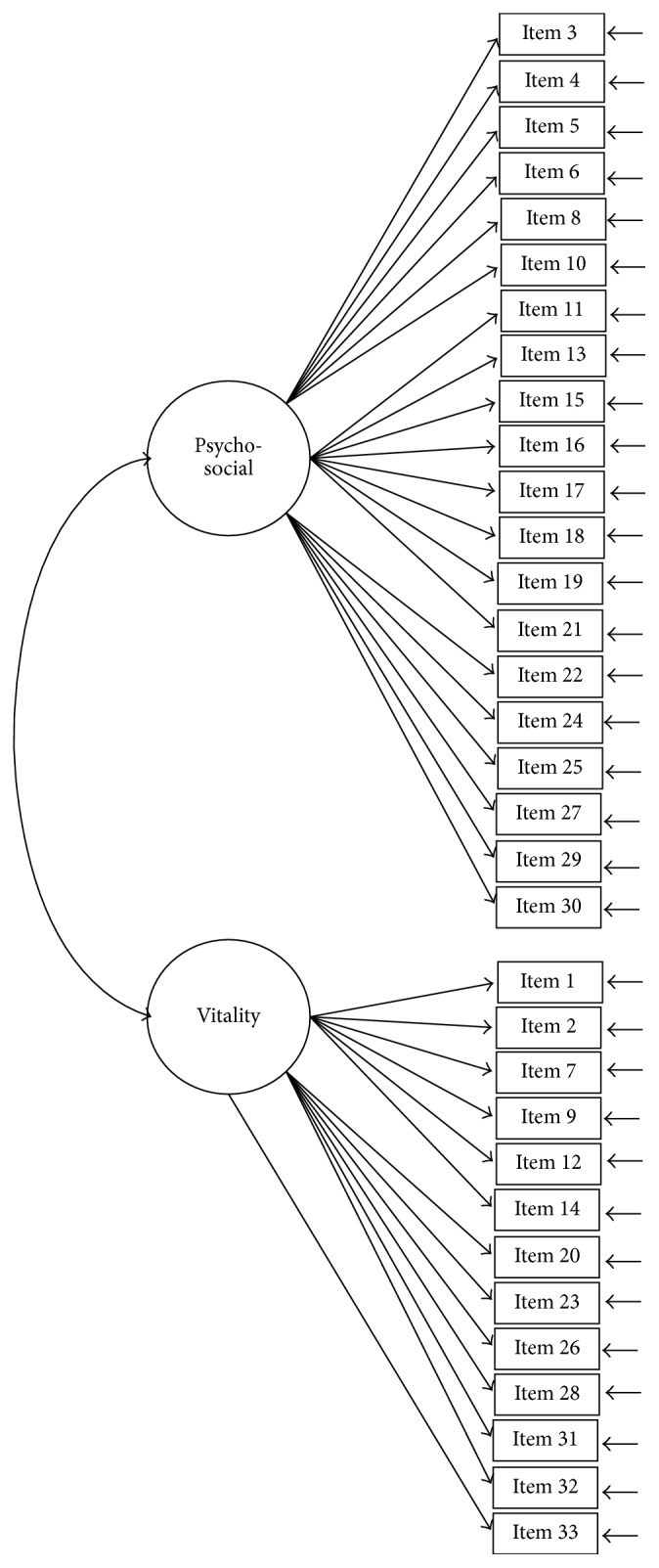
Model 1: the two-correlated-factor model with 33 items. Item descriptions are in Table 3.

**Figure 2:**
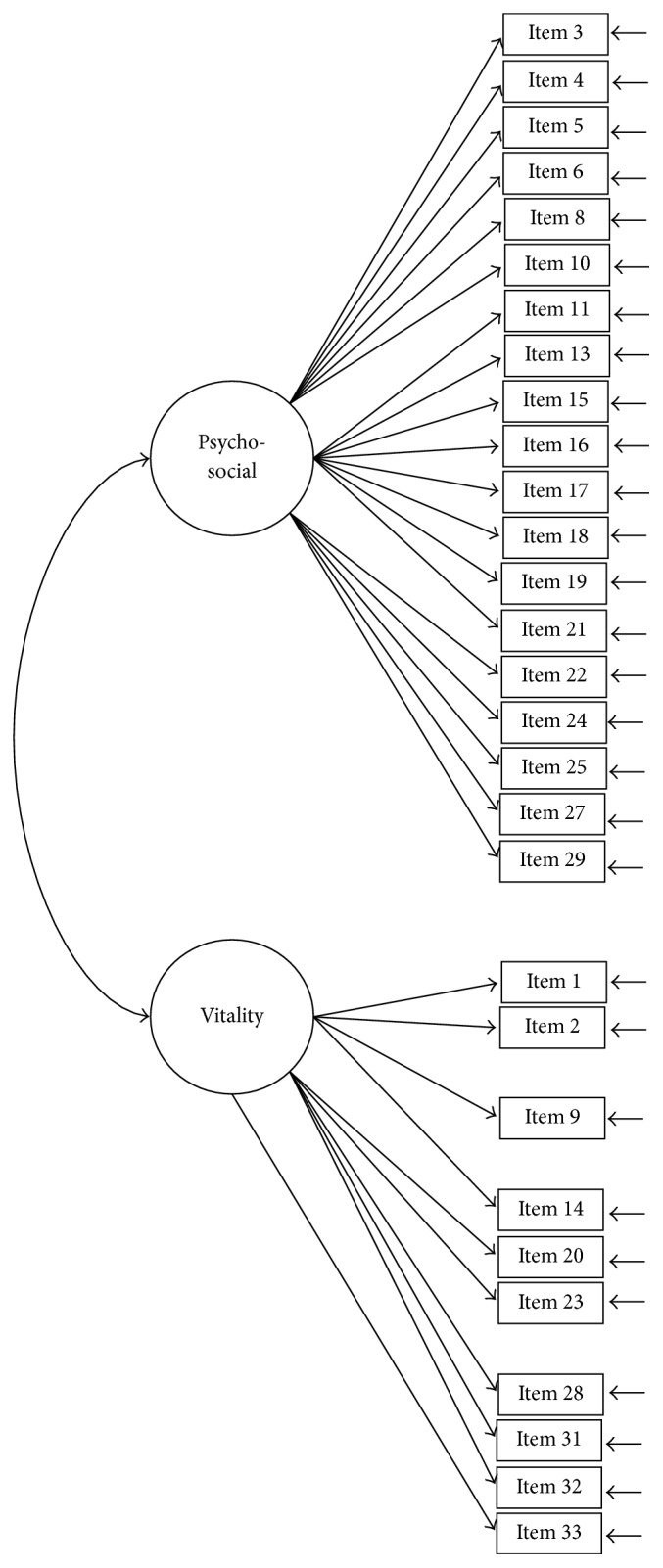
Model 2: the two-correlated-factor model with 29 items. Item descriptions are in Table 3.

**Figure 3:**
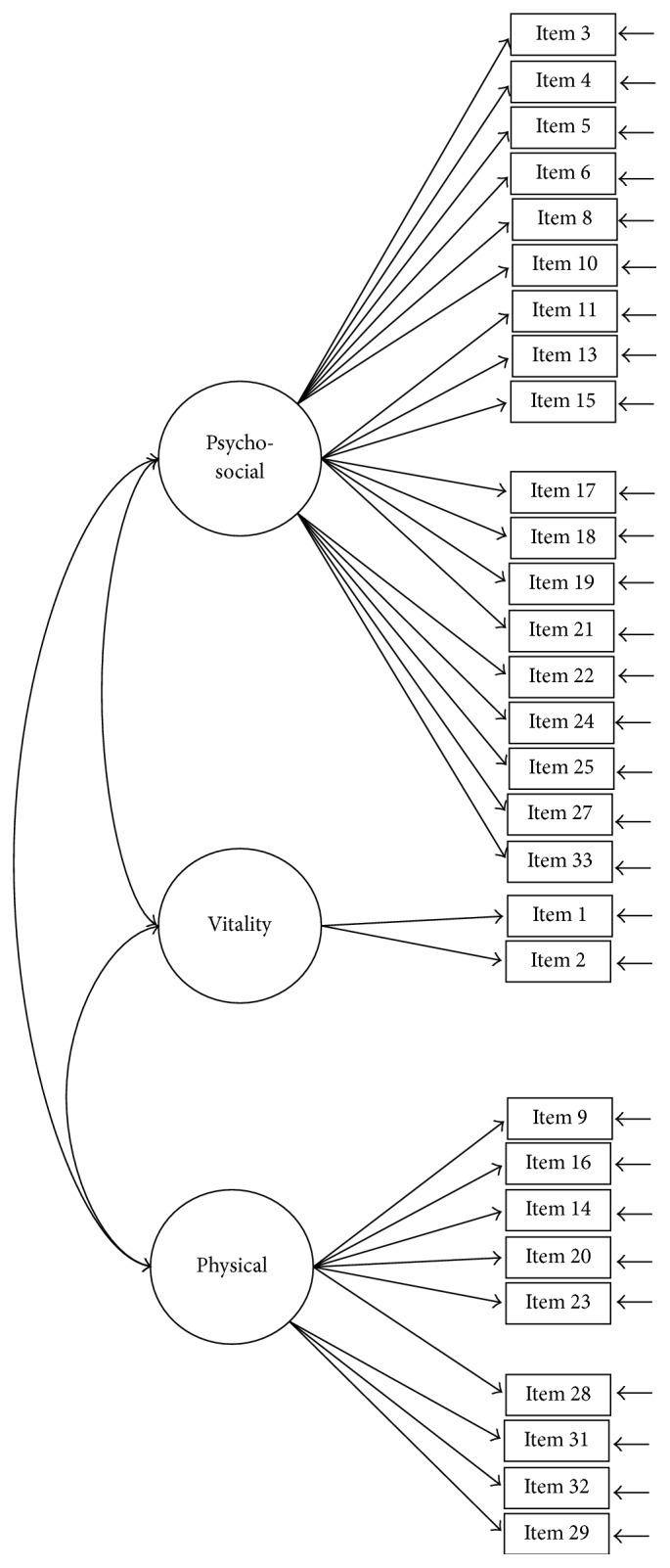
Model 3: the three-correlated-factor model with 29 items. Item descriptions are in Table 3.

**Figure 4:**
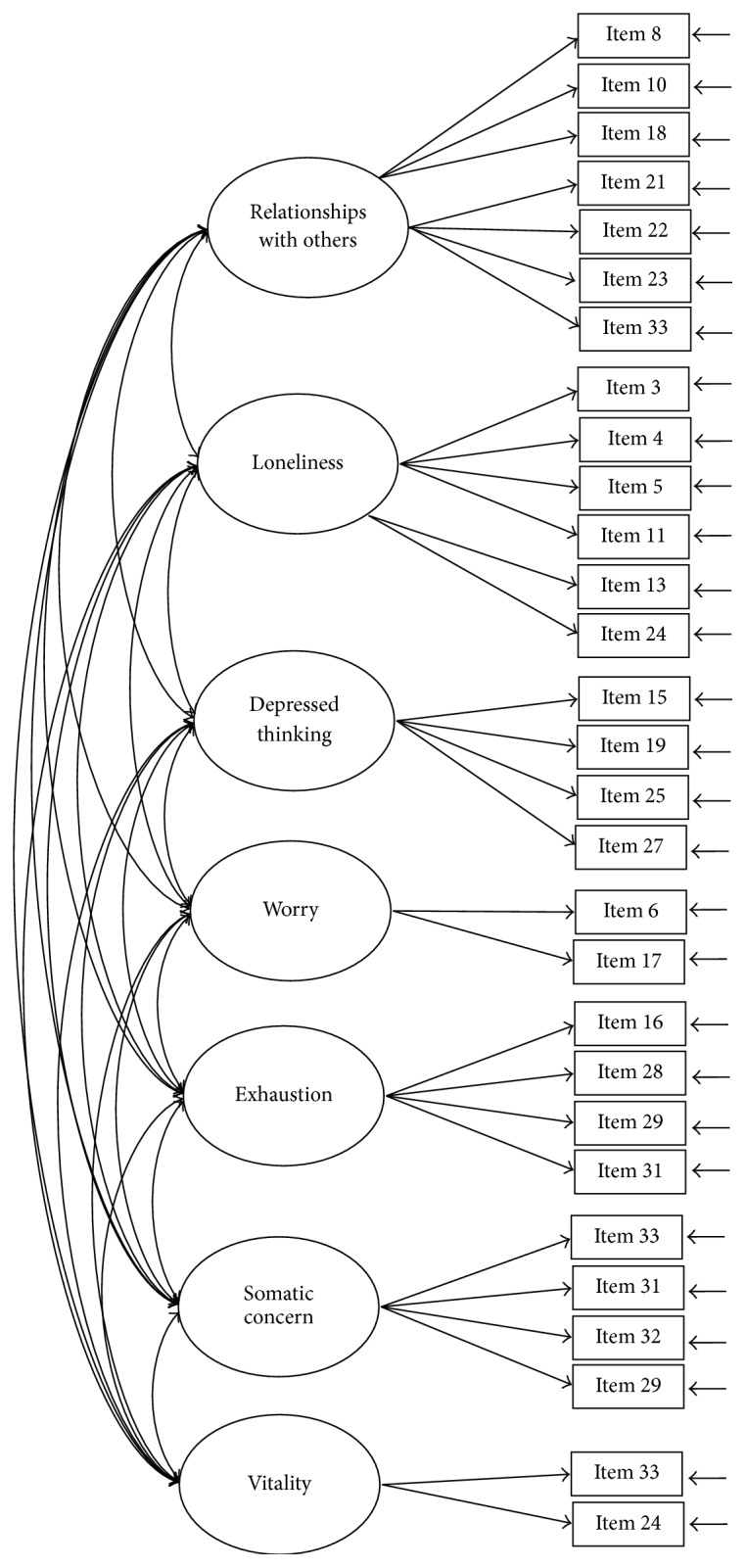
Model 4: the seven-correlated-factor model with 29 items. Item descriptions are in Table 3.

**Figure 5:**
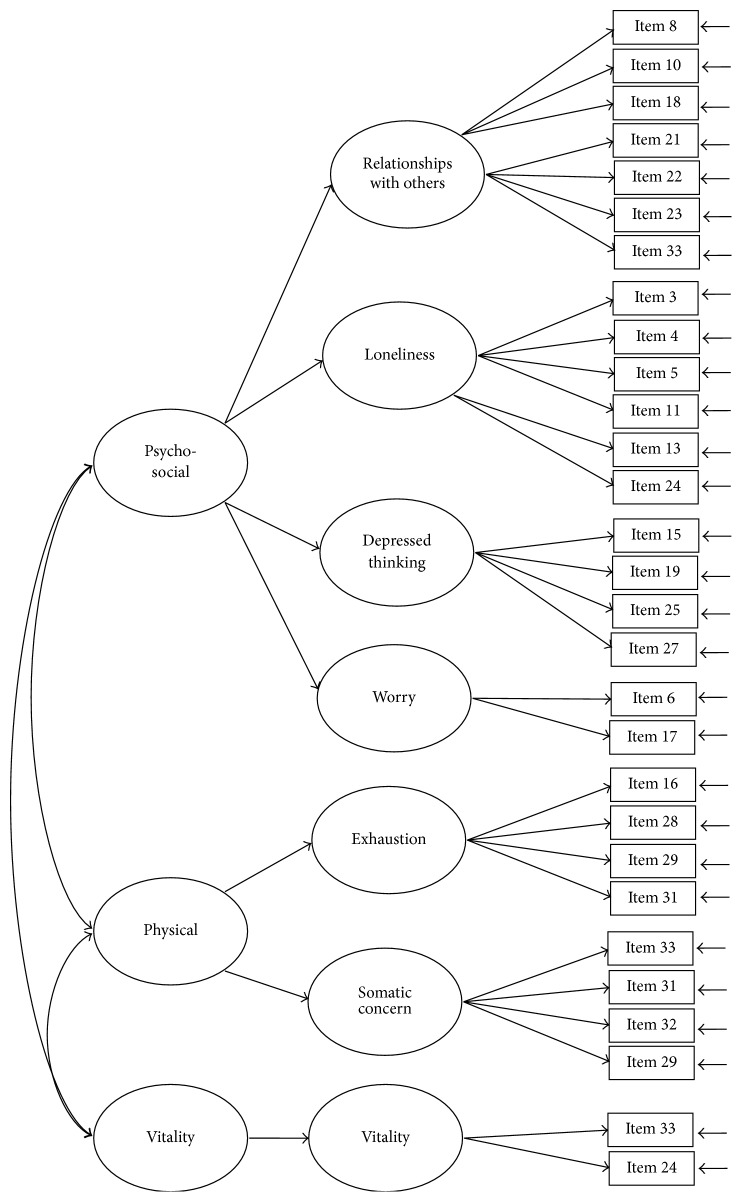
Model 5: the second-order model with 29 items. Item descriptions are in Table 3.

The construct of the SQLS-R4 was examined using CFA models with maximum-likelihood estimations. Five CFA models were tested: Models 1 (33 items; Figure 1) and 2 (29 items; Figure 2) were two-correlated-factor models (psychosocial and vitality). Models 3 and 4 were three-correlated-factor (psychosocial, physical, and vitality; Figure 3) and seven-correlated factor (relationships with others, loneliness, depressed thinking, worry, exhaustion status, somatic concern, and vitality; Figure 4) models, respectively. Unlike Models 1 to 4, all first-order models, Model 5 (Figure 5) was a second-order model with three dimensions correlated in the second-order (psychosocial, physical, and vitality), and seven first-order factors embedded in the second-order factors (psychosocial: relationships with others, loneliness, depressed thinking, and worry; physical: exhaustion status and somatic concern; and vitality: vitality). The underlying factors of the proposed models were correlated based on the suggestions of Martin and Allan [7].

To examine the data-model fit for the five models, we used the *χ*^2^ test and four additional indices: the comparative fit index (CFI), Tucker-Lewis index (TLI), root mean square error of approximation (RMSEA), and standardized root mean square residual (SRMR). The values of the CFI and TLI were > 0.9 [22], and those of the RMSEA and SRMR were < 0.08, which suggests that the data-model fit is acceptable [23, 24]. Moreover, to compare the five models, Akaike's information criterion (AIC) and expected cross-validation index (ECVI) were consulted; smaller values indicate a better fit [24].

After the construct of the SQLS-R4 had been determined, concurrent validity using Pearson correlation was done to strengthen the validity performance of the SQLS-R4. The correlation between each domain of the WHOQOL-BREF and each factor of the SQLS-R4 was tested, and an absolute correlation coefficient > 0.3 suggests a fair correlation [25].

The CFAs were done using Lisrel 8.8 (Scientific Software International, Lincolnwood, IL, USA), and Pearson correlation was done using SPSS 16.0 (SPSS Inc., Chicago, IL, USA).

## 3. Results

The age, age at onset of schizophrenia, duration of schizophrenia, and duration of institutionalization for schizophrenia of the participants are presented in Table 1. Two-thirds of the participants were male, sixty-one percent of them had never been employed, and more than three-quarters of them were single (Table 1).

For the model comparisons, we first determined that the 29-item SQLS-R4 (Model 2: AIC = 685.519; ECVI = 6.924) was a better data-model fit than was the 33-item SQLS-R4 (Model 1: AIC = 994.108; ECVI = 10.041) (Table 2). Thus, the remainder of the models examined (3, 4, and 5) had 29 items. All of Model 3's fit indices were acceptable (CFI = 0.967, TLI = 0.964, RMSEA = 0.068, SRMR = 0.072, AIC = 666.993, and ECVI = 6.737) and outperformed the other models (CFI = 0.928 to 0.963, TLI = 0.918 to 0.960, RMSEA = 0.072 to 0.107, SRMR = 0.073 to 0.132, AIC = 685.519 to 914.583, and ECVI = 6.924 to 9.238). Based on the results, the SQLS-R4 fit the first-order three-dimensional construct the best. In addition, the standardized factor loadings of Model 3 were all significant (Table 3).

The correlation between the SQLS-R4 and the WHOQOL-BREF was then examined. All the factors underlying the SQLS-R4 were significantly and fairly correlated with the dimensions in the WHOQOL-BREF (all *P*s < 0.01 and absolute *r* > 0.3) except for the correlation between vitality and psychological (*r* = −0.26, *P* < 0.01) (Table 4).

## 4. Discussion

In this study, we examined the construct of a newly developed, schizophrenia-specific self-reported QoL measure (SQLS-R4) for people with schizophrenia in Taiwan. To the best of our knowledge, this is the first study that compares several possible factor structures of the SQLS-R4 using CFA and that confirms the construct of the SQLS-R4 for the Asian population. The proposed three-factor model (psychosocial, physical, and vitality) fit best with our data. Moreover, this proposed model was supported by the correlation between its 3 factors on the SQLS-R4 and the factors of the WHOQOL-BREF.

Based on our belief that the underlying concepts should all be correlated because of their relevance to QoL, we did not examine the uncorrelated-factor models. Similar findings were concluded by Martin and Allan [7], who proposed three-factor structures (namely, a one-factor structure, a two-correlated-factor model, and a two-uncorrelated-factor model) and found that the two-correlated factor model had the best data-model fit. However, they also reported that the two-correlated-factor model was not good enough. Therefore, in our study, three more factor structures were compared, and we found that the three-correlated-factor model (psychosocial, physical, and vitality) was the best construct for the SQLS-R4. In addition, our results also suggested that four items on the SQLS-R4 (i.e., Item 7 [able to carry out daily activities]; Item 12 [feel I can cope]; Item 26 [feel happy]; and Item 30 [concerned about social life]) can be omitted. Items 26 and 30 are new to the SQLS-R4 and have relatively low factor loadings (<0.3) in the vitality construct and psychosocial construct, in previous studies [6, 13]. The wordings of these two new items may still be under development, which might contribute to their unstable psychometric properties [24]. Another possible reason is that the participants in our study were institutionalized and may have had limited social lives as well as happy feelings. Thus, further studies may test these two items for people with schizophrenia who live in communities to verify our hypothesis. Items 7 and 12 were found to have higher correlations with the total score of the motivation and energy domain in the SQLS for the UK version (*r* = 0.67 and 0.68) [8] and for English-speaking Asians (*r* = 0.51 and 0.42) [11] than for the Japanese version (*r* = 0.39 and 0.34) [10] and for Chinese-speaking Asians (*r* = 0.21 and 0.32) [11]. Therefore, the two items may be perceived differently from the vitality construct in Asian and Western cultures [6, 12].

The factor structure of the SQLS/SQLS-R4 has been revised several times [9], and its psychometric properties have been tested (e.g., Wilkinson et al. [8]; Kaneda et al. [10]; and Kuo et al. [13]). Three methods have been used: concurrent validity [6, 10–12, 26], exploratory factor analysis [8, 13], and CFA [7]. Except for the CFA, concurrent validity and exploratory factor analysis indirectly tested the construct of the SQLS-R4. Concurrent validity is used to examine the relationship between several measures [27] (e.g., the relationship between the SQLS-R4 and the WHOQOL-BREF). Because the constructs of other measures (e.g., the WHOQOL-BREF) are not the same as that of the SQLS-R4, the SQLS-R4 construct cannot be precisely examined using concurrent validity. Exploratory factor analysis is used to explore the possible underlying factors of one measure [28], such as the SQLS-R4; it was suitable for identifying a set of latent constructs underlying the measured items on the SQLS-R4 without an a priori hypothesis factor structure. In other words, exploratory factor analysis explores rather than examines the construct of SQLS-R4. Therefore, we suggest that it would be more appropriate to compare our results with those of Martin and Allan [7], who also used CFA to examine the factor structure of the SQLS-R4.

The main difference between the best-fit structure of the SQLS-R4 in this study and the former structure of the SQLS-R4 is one construct (physical) added to our suggested structure. Although the structure of the SQLS-R4 is designed without a physical construct [8, 9], the definition of QoL includes the concept of physical performance [1, 4, 29]. Therefore, the construct of physical should also be emphasized for the SQLS-R4, as Kuo et al. [13]. have recommended. The physical construct is defined as an individual's ability to perform daily activities (e.g., self-care; rest) and is related to one's physical condition [29]. Thus, some items on the SQLS-R4 (e.g., physically weak; slept well; restless) are suitable for the physical construct, and they were verified in a report on health-related quality of life measures [29]. Because no published studies have examined the three-correlated-factor model of the SQLS-R4 for any Western population, we suggest that our three-correlated-factor model should be tested using Western populations to further understand the physical construct.

In addition to the CFA-based confirmation of the factor structure of the SQLS-R4, our concurrent validity test also supported the SQLS-R4 construct. Significantly moderate-to-high correlations were found between the SQLS-R4 constructs and the WHOQOL-BREF constructs, except for a low correlation between the vitality construct of the SQLS-R4 and the psychological construct of the WHOQOL-BREF (*r* = −0.26). Consistent with our findings, Chou et al. [30]. also found a nonsignificant correlation between the vitality construct of the SQLS-R4 and the psychological construct of the S-QoL. The content of vitality construct items (namely, “lack energy” and “couldn't be bothered”) seems to describe more about physical condition and social interaction than about mental function, which may contribute to the low correlation.

This study has several limitations. There were only 100 participants in this study, the minimum required for a reliable CFA [16, 17]. However, because the replication of a factor structure is recommended for CFA [16], our findings for the SQLS-R4 construct can be seen as a preliminary outcome for the Asian population. Future studies may conduct other CFAs based on our findings to strengthen the construct of the SQLS-R4. Second, all the participants were inpatients from one psychiatric hospital; thus, our findings may not be generalizable to people with schizophrenia and living in the community. Third, we recruited only participants with intact cognitive ability (MMSE ≥ 24 and BPRS < 24); therefore, the SQLS-R4 construct we suggested in this study cannot be generalized to those with impaired cognitive ability.

In conclusion, our results suggested a three-correlated-factor construct of SQLS-R4 for people with schizophrenia in Taiwan. In addition, the reliability and validity were good for the SQLS-R4. However, because our participants were all institutionalized, additional studies that examine the SQLS-R4 construct on outpatients with schizophrenia are needed. Moreover, comparing the SQLS-R4 construct for Asian and Western populations is also suggested.

## Figures and Tables

**Table 1:** Participants' characteristics.

Variable	*n* or mean ± SD
Gender	
Male	66
Female	34
Education level	
Junior high school or below	44
Senior high school or above	56
Previously or currently employed?	
Yes	39
No	61
Marital status	
Single	76
Married	10
Other	14
Age (year)	49.2 ± 7.8
Age at onset of schizophrenia (year)	22.6 ± 5.8
Duration of schizophrenia (year)	26.6 ± 8.1
Duration of institutionalization for schizophrenia (year)	17.4 ± 8.7

**Table 2:** Model comparisons.

	Model number				
	1	2	3	4	5
*χ* ^2^ (*df*)	860.108 (494)	567.519 (376)	544.993 (374)	756.583 (356)	675.982 (368)
CFI	0.934	0.963	0.967	0.928	0.945
TLI	0.929	0.960	0.964	0.918	0.939
RMSEA	0.087	0.072	0.068	0.107	0.092
SRMR	0.090	0.073	0.072	0.132	0.074
AIC	994.108	685.519	666.993	914.583	809.982
ECVI	10.041	6.924	6.737	9.238	8.182

Model 1: two correlated factors (psychosocial and vitality) with 33 items.

Model 2: two correlated factors (psychosocial and vitality) with 29 items.

Model 3: three correlated factors (psychosocial, physical, and vitality) with 29 items.

Model 4: seven correlated factors (relationships with others, loneliness, exhaustion, depressed thinking, somatic concern, vitality, and worry) with 29 items.

Model 5: seven correlated factors (relationships with others, loneliness, exhaustion, depressed thinking, somatic concern, vitality, and worry) underlying three correlated dimensions (psychosocial, physical, and vitality) with 29 items.

CFI = comparative fit index; TLI = Tucker-Lewis index; RMSEA = root mean square error of approximation; SRMR = standardized root mean square residual; AIC = Akaike's information criterion; ECVI = expected cross-validation index.

**Table 3:** Standardized factor loadings on SQLS-R4 with three underlying constructs.

Item number	Item description	Standardized factor loadings		
		Psychosocial	Physical	Vitality
1	Lack energy	—	—	0.816
2	Couldn't be bothered	—	—	0.769
3	Worry about future	0.475	—	—
4	Lonely	0.574	—	—
5	Hopeless	0.654	—	—
6	Panicky	0.632	—	—
7	Able to carry out daily activities^a^	—	—	—
8	Took things people said the wrong way	0.552	—	—
9	Hard to concentrate	—	0.606	—
10	Difficult to mix with people	0.536	—	—
11	Down	0.753	—	—
12	Feel I can cope^a^	—	—	—
13	Mixed up and unsure	0.662	—	—
14	Slept well	—	0.261	—
15	Have mood swings	0.769	—	—
16	Concerned wouldn't get better	—	0.554	—
17	Worry	0.569	—	—
18	People avoid me	0.684	—	—
19	Upset about past	0.537	—	—
20	Poor memory	—	0.644	—
21	Cut off from world	0.543	—	—
22	Uncomfortable with people	0.740	—	—
23	Can't think clearly	0.512	—	—
24	Upsetting thoughts	0.766	—	—
25	Suicidal thoughts	0.562	—	—
26	Feel happy^a^	—	—	—
27	Depressed	0.740	—	—
28	Drowsy	—	0.654	—
29	Restless	—	0.577	—
30	Concerned about social life^a^	—	—	—
31	Tired	—	0.647	—
32	Physically weak	—	0.637	—
33	Wasn't leading normal life	0.604	—	—

All *P*s < 0.01

^a^Items not in the three-correlated-factor model.

**Table 4:** Correlations between the SQLS-R4 and the WHOQOL-BREF.

	SQLS-R4		
	Psychosocial	Physical	Vitality
WHOQOL-BREF			
Physical	−0.50	−0.64	−0.39
Psychological	−0.41	−0.53	−0.26
Social	−0.38	−0.48	−0.38
Environment	−0.59	−0.69	−0.52

All *P*s < 0.01.
